# Purification and structural elucidation of three bioactive compounds isolated from *Streptomyces coelicoflavus* BC 01 and their biological activity

**DOI:** 10.1007/s13205-016-0581-9

**Published:** 2017-04-11

**Authors:** Kothagorla Venkata Raghava Rao, Palla Mani, Botcha Satyanarayana, Tamanam Raghava Rao

**Affiliations:** 1 Department of Biochemistry, Andhra University, Visakhapatnam, Andhra Pradesh 530 003 India; 2 Department of Organic Chemistry, Andhra University, Visakhapatnam, 530 003 India

**Keywords:** Fermentation, *Streptomyces coelicoflavus* BC 01, Chromatography, Antioxidant, Anti-inflammatory

## Abstract

The strain *Streptomyces coelicoflavus* BC 01 was isolated from mangrove soil and used as inoculum for submerged fermentation. The fermented broth was extracted with ethyl acetate, the crude extract was subjected to silica gel column chromatography and the homogeneity of the isolated fractions was determined by TLC and then subjected to RP-HPLC for their purity. The purification steps led to the isolation of three pure bioactive compounds named as BC 01_C1, BC 01_C2 and BC 01_C3. The chemical structure of these three compounds was established on the basis of their spectroscopic studies like UV, IR, ^1^H and ^13^C NMR and GC–MS data by comparison with reference data from literature. The structure of the compound BC 01_C1 was established as 5-amino-2-(6-(2-hydroxyethyl)-3-oxononyl) cyclohex-2-enone. The compound BC 01_C2 was established as8-(aminomethyl)-7-hydroxy-1-(1-hydroxy-4-(hydroxylmethoxy)-2,3-dimethylbutyl)-2-methyl dodecahydro phenanthren-9(1H)-one and the compound BC 01_C3 was established as1-((E)-2-ethylhex-1-en-1-yl)2-((E)-2-ethylidenehexyl)cyclohexane-1,2-dicarboxylate. The MIC values of the three isolated compounds (BC 01_C1, BC 01_C2 and BC 01_C3) were found between 12.5–75 μg/ml for bacteria and 50–125 μg/ml for fungi used in this study. These compounds also possess in vitro antioxidant and anti-inflammatory activities.

## Introduction

The development of resistance to multiple drugs is a major problem in the treatment of infections by pathogenic microorganisms. This antimicrobial resistance is presently an urgent focus of research and novel bioactive compounds are necessary to combat these pathogens. Microbial natural products are the most important source of new medicines. Among the potential sources of natural products bacteria are very important (Newman and Cragg 2007). However, the ability to produce anti-infective agents is limited to 5 of the 53 known phyla of bacteria. The most productive are members of the class actinobacteria; approximately 7000 compounds reported in the dictionary of natural products are of actinobacterial origin. Therefore, pharmaceutical industries are mainly focused on production of actinomycetes because of their ability to produce secondary metabolites (Berdy 2005). The secondary metabolites are organic compounds that are not directly involved in the normal growth, development or reproduction of the producing organism (Martín et al. 2005). The secondary metabolites are secreted during the generation of aerial hyphae from the vegetative mycelium (Maguelez et al. 2000). The medical uses of these secondary metabolites are not just limited to antibiotics but also include antibacterial, antifungal, antiviral, anticancer, antiparasitics, immunosuppressant agents, insecticides, herbicides, vitamins and enzymes, etc. (Newman and Cragg 2007). Other secondary metabolites produced by actinomyctes such as aliphatic alcohols, lactones, biogenic sulphides, ketones, esters, thioesters, lactones, furanones and isoprenoids are important to the chemical and pharmaceutical industries (Zaitlin and Watson 2006).

The genus *Streptomyces* is an economically important group of organisms among actinomycetes and they are the key source for wide range of biologically active compounds (Berdy 2005). About three-quarters of all the known commercially and medicinally useful antibiotics and several agriculturally important compounds were obtained from the *Streptomyces* sp. (Cundlife 1989). Furthermore, approximately 60% of the antibiotics discovered in the year 1990 and most of the antibiotics used in agriculture are from the genus *Streptomyces.* They have the ability to synthesize many biological activity compounds; hence they are extensively used as an important research subject from the academicians and as well as industrialists (Tanaka and Mura 1993). The present study describes the extraction, purification and the structural elucidation of three bioactive compounds from a liquid culture broth of the strain *Streptomyces coelicoflavus* BC 01 which was isolated from mangrove soil of Visakhapatnam, Andhra Pradesh, India. The biological activities of these three compounds are also addressed in the study.

## Materials and methods

### Isolation and maintenance

The sediment samples were collected from mangrove environment (16°–30′ to 17°–00′N latitudes and 82°–14′ to 82°–23′E longitudes) near Dockyard, Visakhapatnam. The pH of the soil is around 7.6–8.0 and the salinity of the soil is of sandy texture. The soil sample was collected from 5 to 10 cm depth during the months of April–June 2010 by inserting sterile corer into the soil. Samples were transferred to a sterile polythene bag and stored under aseptic conditions until further use. The strain *S. coelicoflavus* BC 01 was previously isolated from the soil sample collected at mangrove area in Visakhapatnam, Andhra Pradesh, India (Rao and Rao 2013). This strain was isolated by soil dilution plate technique using yeast extract malt extract glucose agar (ISP 2) media with the addition of 2.5 mg/ml of rifampicin and 75 mg/ml of fluconazole to inhibit unwanted bacterial and fungal contaminations, respectively. The strain was sub-cultured on starch casein agar medium incubated at 28 °C for 5–7 days to achieve good sporulation and was preserved at 4 °C in refrigerator for further use.

### Inoculum preparation of *Streptomyces coelicoflavus* BC 01

The isolate *S. coelicoflavus* BC 01 was used in the present study. 5 ml of sterile 0.9% NaCl solution was added to 7-day-old well-sporulated slant of the culture. The spores were scraped from the slant into sterile saline solution and the resulting spore suspension (10%) was transferred aseptically into a 500-ml Erlenmeyer flask containing 200 ml of inoculum medium. The inoculum medium comprises (g/L) glucose 10.0, soya bean meal 10.0, NaCl 10.0 and CaCO_3_ 5.0 with pH 7.0. The inoculated flasks were kept in an orbital shaker (120 rpm) at 28 °C for 48 h. The contents of the flasks were centrifuged at 3000 rpm for 10 min and the supernatant discarded. The cell pellet was washed thoroughly and suspended in 0.9% NaCl solution. This cell suspension was used as inoculum.

### Submerged fermentation process for antibiotic production

The submerged fermentation (6 L) was conducted by shake flask method in 500 ml Erlenmeyer flasks. A 10% (v/v) of 48 h aged inoculum was transferred to 200 ml of production medium having composition glucose 12.0 g/L; Soya bean meal 10.0 g/L; K_2_HPO_4_ 2.5 g/L, NaCl 10.0 g/L; trace salt solution 1.0 ml {(CuSO_4_·5H_2_O (0.64 g/L); FeSO_4_·7H_2_O (0.11 g/L); MnCl_2_·4H_2_O (0.79 g/L); ZnSO_4_·7H_2_O (0.15 g/L)} with pH of the medium 7.2 in 500 ml Erlenmeyer flasks (30 flasks were used, each flask containing 200 ml of the production medium). All the flasks were incubated on a rotary shaker (160 rpm) at 30 °C for 96 h and then harvested. After harvesting, the dark pink colour culture broth (6 L) was filtered using Whatmann No. 1 filter paper to separate mycelium from the liquid phase. The mycelial cake was centrifuged at 4000 rpm for 10 min and a clear culture filtrate separated.

### Extraction of crude antibiotic from fermented broth

The solvent extraction was used for the first step in the whole separation process. The clear culture filtrate (1 L) was extracted twice with ethyl acetate of 1:1 (v/v) and shaken vigorously for 1 h for complete extraction. The ethyl acetate phase that contains bioactive compound was separated from the aqueous phase. It was evaporated to dryness under reduced vacuum 80°–90 °C. The obtained crude ethyl acetate extract was subjected to column chromatography.

### Purification of compounds from crude ethyl acetate extract by column chromatography

Silica gel of l00–200 µm particle size was selected for column chromatography. The silica gel was suspended in chloroform for packing the column. The column consisted of a 40-cm-long corning glass tube having an internal diameter of 2.5 cm with a glass stopper at the bottom. The lower end of the tube contained stinted disc and the washed silica gel suspension was introduced gradually to obtain an air-bubble free continuous column. The final size of the column was 25 × 2.5 cm. The column was equilibrated with chloroform. The sample not exceeding 5 ml was passed through the column keeping the flow rate at 0.2 ml/min. with gradient solvent system consisting of chloroform: methanol (9:1, 7:3, 1:1). Finally the column was washed with methanol. Fractions of 5 ml with each solvent system were collected and all the individual fractions were analysed by TLC for homogeneity.

### Determination of homogeneity of the fractions

The homogeneity of the isolated fractions was determined by Reverse Phase partition TLC using polar phase solvent system chloroform: methanol and non-polar phase solvent system 5% (v/v) n-hexane in petroleum ether. TLC was carried out on silica gel 60 F_254_ plates (Merck, 0.25 mm) impregnated with non-polar phase by immersing the activated plate into a solution of the non-polar phase and allowing the solvent to evaporate. Approximately 10 µl of the each fraction was applied with a capillary tube. The chromatograms were run and the spots on the TLC plates were visualized by keeping them in a closed chamber containing iodine vapours. Fractions with similar R_f_ values were pooled together and evaporated to dryness under reduced vacuum to check their purity.

### Checking the purity of compounds by reverse-phase high-performance liquid chromatogram (RP-HPLC)

HPLC is a very popular method and is widely used to separate and quantify compounds for the identification and isolation of bioactive natural products. The purity of the antibiotic compounds was analysed by analytical HPLC supported with Waters Spherisorb 5 μm ODS2 4.6 X 250 mm analytical cartridge (C-18 column) on a Waters 515 pump; isocratic Reverse phase system with a 2998 photodiode array detector at 210 nm and the range given was 190–600 nm. The flow rate was 1.0 ml/min, and additional UV detector was measured at 254 nm using Empower 2 software. Methanol was used as mobile phase. The purified compounds were mixed with HPLC grade methanol and filtered by using 0.22 μ Millipore membrane filter before injecting into injection port. The samples were run for 15 min and the retention time was noted; based on the percentage of area of the peak the purity of the compound was known.

### Structural elucidation of the antibiotic compounds

#### Solubility

The solubility pattern of the compound was determined in various polar and non-polar solvents.

#### Melting point

The melting points were recorded on Kumar capillary melting point apparatus and are uncorrected.

#### UV-absorption spectrum

Ultraviolet (UV) spectrums were recorded on Shimadzu UV-1800 spectrophotometer. The compounds obtained from *S. coelicoflavus* BC 01 were dissolved in methanol at a concentration of 1 mg/ml and the spectrums were recorded at 200–500 nm range using UV-Probe software.

#### FT-IR spectrum

The infrared spectra were recorded on Shimadzu IR-470 model. The spectra were scanned in the range of 750–3500 cm^−1^. The spectra were obtained using potassium bromide pellet technique. The spectra were plotted as intensity versus wave number. The FT-IR spectra were analysed for the presence of functional groups in pure compounds.

#### Mass spectrum

The purified compounds were analysed on Agilent GC–MS system (GC: 5890 series II; MSD 5972). The fused-silica HP-5 capillary column (30 m × 0.25 mm, ID, film thickness of 0.25 mm) was directly coupled to the MS. The carrier gas was helium with a flow rate of 1.2 ml min^−1^. Oven temperature was programmed (50 °C for 1 min, then 50–280 °C at a rate of 5 °C/min) and subsequently held isothermally for 20 min. The temperature of injector port was maintained at 250 °C and that of detector at 280 °C. The peaks of the obtained components in gas chromatography were subjected to mass-spectral analysis.

#### ^1^H and ^13^C NMR

The nuclear magnetic resonance (NMR) spectra of purified compounds were recorded on 400 MHz Fourier transform nuclear magnetic resonance (Bruker Model: Avance-II) spectrophotometer. The chemical shifts were expressed in *δ* (ppm) using CDCl_3_ as solvent and trimethylsilane (TMS) as internal reference.

### Biological activities of the purified compounds

#### Minimum inhibitory concentration (MIC)

Different concentrations of the purified compounds (12.5, 25, 50, 75, 100, 125, 150, 175, 200 and 250 µg/ml) were tested for their minimum inhibitory concentration (MIC) against bacteria and fungi by using agar well diffusion method (Wiegand et al. 2008). The results were expressed as the minimum concentration of the compound for total inhibition against bacterial or fungal growth. The test organisms procured from IMTECH, Chandigarh and used for the determination of minimum inhibitory concentration (MIC)were *S. aureus* (MTCC 3160), *B. subtilis* (MTCC 441), *B. cereus* (MTCC 430), *P. aeruginosa* (MTCC 424), *E. coli* (MTCC 443), *P. vulgaris* (MTCC 426), *C. albicans* (MTCC 227), *A. niger* (MTCC 961), *A. flavus* (MTCC 3396) and *S. cerevisiae* (MTCC 170).

#### DPPH (1, 1, diphenyl-2-picryl hydrazyl) scavenging activity

DPPH was assayed by the method of Parasuraman et al. (2010) with slight modifications. Different concentrations (5, 10, 15 and 20 µg/ml) of above compounds and crude were dissolved in methanol and taken in test tubes separately. Ascorbic acid was used as a reference standard. DPPH 0.004% was freshly prepared in methanol. DPPH (2 ml) was added to each tube containing different concentrations of compounds (1 ml) and of standard solution (1 ml). It was shaken vigorously. They were then allowed to stand for 30 min at room temperature in dark place. The control was prepared without any compound. Methanol was used for base line corrections and absorbance (OD) of sample was measured at 517 nm. The below formula was used to interpret the value of the sample.

$$\% {\text{ Radical scavenging activity }} = \, \left[ {\left( {{\text{Control O}}.{\text{D }}{-}{\text{ sample O}}.{\text{D}}} \right) \, /{\text{ Control O}}{\text{D}}}\right] \, \times { 1}00$$

#### Ferric reducing antioxidant power assay (FRAP)

FRAP assay was carried out according to the method of Benzie and Strain (1996). FRAP assay uses antioxidants as reductants in a redox-linked colorimetric method, employing an easily reduced oxidant system present in stoichiometric excess. The FRAP reagent was prepared by mixing 300 mM acetate buffer (pH 3.6), 10 mM TPTZ and 20 mM FeCl_3_·6H_2_O in a ratio of 10:1:1, at 37 °C. 1.5 ml of the FRAP reagent was mixed with 0.5 ml of the sample (compound) and the absorbance was measured at 593 nm after 15 min. The results are expressed in µmoles/ml of ascorbic acid equivalents.

#### Determination of total antioxidant capacity

The total antioxidant capacity was evaluated by the phosphomolybdenum method according to the procedure described by Prieto et al. (1999). The assay is based on the reduction of Mo (VI)–Mo (V) by the extract and subsequent formation of a green phosphate/Mo (V) complex at acid pH. 0.1 ml of sample was taken in methanol, combined with 1.9 ml of reagent solution (0.6 M sulphuric acid, 28 mM sodium phosphate and 4 mM ammonium molybdate). The tubes were capped and incubated at 95 °C for 90 min. After the samples were cooled to room temperature, the absorbance was measured at 695 nm against a blank. Ascorbic acid equivalents were calculated using standard graph of ascorbic acid and values are expressed as ascorbic acid equivalents in µg/mL of extract.

#### In vitro anti-inflammatory activity

The human red blood cell (HRBC) membrane stabilization method has been used to study the in vitro anti-inflammatory activity (Gandhidasan et al. 1991). Fresh blood sample was collected from healthy individual at the Andhra University dispensary and mixed with equal volume of sterilized Alsever solution (2% dextrose, 0.8% sodium citrate, 0.05% citric acid and 0.42% sodium chloride in distilled water).The blood was centrifuged at 3000 rpm and packed cells were washed with isosaline (0.85%, pH 7.2) and a suspension was made with isosaline (10% v/v).

#### Membrane stabilization assay

The assay mixture contained 1 ml of phosphate buffer (0.15 M, pH 7.4), 2 ml of hyposaline (0.36%), 0.5 ml of HRBC suspension and 1 ml of various concentrations of the test compound. Diclofenac sodium was used as reference drug. In the control solution, instead of hyposaline, 2 ml of distilled water was added. The mixtures were incubated at 37 °C for 30 min and centrifuged. The absorbance of the supernatant solution was read at 560 nm. The percentage of haemolysis was calculated by assuming the haemolysis produced in the presence of distilled water as 100%. The percentage of HRBC membrane stabilization was calculated using the following formula:

$${\text{Percentage of membrane stabilization }} = \frac{{ 100 - {\text{OD of drug treated sample}}}}{\text{OD of control}} \times { 1}00$$

### Statistical analysis

The structure of the compounds was drawn using ChemBioOffice Version 12. All investigations were conducted in triplicate and the attained data were subjected to one-way ANOVA using SPSS version 16 and the significance level was 0.05. The obtained data were expressed as mean ± standard error.

## Results and discussion

In microbial community mangrove actinomycetes become a hot spot for isolation of natural products. Currently, 122 various secondary metabolites were isolated from mangrove actinomycetes; of these, 73 compounds were novel and remaining compounds were predicted previously (Xu et al. 2014). It has long been known that some of the *Streptomyces* strains of the similar species might produce different antibiotics, whereas certain other strains belonging to different species produced the identical antibiotics (Lechevalier 1975). Therefore, the production of antibiotics by *Streptomyces* could not be species-specific, but slightly strain-specific. Antibiotics of *Streptomyces* origin evidence a wide variety of chemical structures, including aminoglycosides, anthracyclines, glycopeptides, β-lactams, macrolides, nucleosides, peptides, polyenes, polyketides, actinomycins and tetracyclines (Baltz 1998). In the present study the isolate *Streptomyces coelicoflavus* BC 01 was extracted using ethyl acetate. The ethyl acetate extract was then evaporated to dryness under reduced vacuum at 80–90 °C in a rotary evaporator. The crude extract appeared as pink-coloured solid material and about 5 g was obtained from the culture filtrate (6 l).

### Purification of compounds from crude ethyl acetate extract by column chromatography

The ethyl acetate extract was purified using silica gel column chromatography. About 5 g of the crude ethyl acetate extract was chromatographed on silica gel column and eluted with gradient solvent system consisting of 100% chloroform: methanol in the ratio of 9:1, 7:3, 1:9 and followed by 100% methanol. A total of 120 fractions of 5 ml each were collected. The purity of all the fractions was analysed by TLC. The fractions with similar Rf values were pooled together, which ultimately resulted in three major fractions. Each of these fractions was evaporated to dryness under reduced vacuum. The obtained fractions were named as fraction I (360 mg), fraction II (526 mg) and fraction III (230 mg). Fraction I was obtained with eluent by 100% chloroform and fraction II was obtained with eluent 9:10 CHCl_3_:CH_3_OH, whereas fraction III by 100% methanol. These three fractions were designated as BC 01_C1, BC 01_C2 and BC 01_C3. All these fractions were characterized by spectral analysis and also tested for their minimum inhibitory concentration against the test organisms, in vitro antioxidant and anti-inflammatory activity.

### Structural elucidation of purified compounds

#### Compound BC 01_C1

**Fig. 1:**
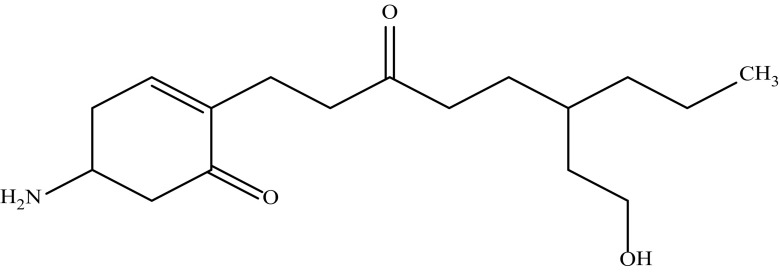
Structure of the compound BC 01_C1

The compound BC 01_C1 was isolated as a pink-colour amorphous powder soluble in methanol, chloroform, ethyl acetate, diethyl ether, ethanol, acetone, benzene, DMSO and insoluble in hexane. The melting point of the compounds was found to be 275 °C. The purity of the compound BC 01_C1was evidenced by HPLC and found to be 99.5%. The UV spectrum of the purified compound BC 01_C1 was dissolved in methanol and the spectrum displayed. Absorption maxima (*λ*_max_) at 207 nm indicate the presence of cyclohexenone ring and *λ*_max_ at 273 nm indicates the presence of keto group, respectively. ^1^H NMR (CDCl_3_: TMS 400 MHz) the spectrum of the compound showed *δ* 0.9 (CH_3_ proton), *δ* 1.25–1.28 (CH_2_ proton adjacent to CH_3_ group), *δ* 1.48–1.49 (CH proton), *δ* 2.04–2.36 (CH_2_ proton), *δ* 3.64 (O–H proton), *δ* 5.34–5.35 (N–H proton), *δ* 6.9 (H–C=C proton). ^13^CNMR (CDCl_3_: TMS 400 MHz) *δ* 14.3 (CH_3_ carbons), *δ* 22.8 (CH_2_ carbon adjacent to CH_3_ group), *δ* 30.37–34.14(CH_2_ carbon), *δ* 36.8–39.2(CH_2_ carbon adjacent to C=O group), *δ* 65.2 (CH_2_ carbon adjacent to OH group), *δ* 130.1–130.2 (olefimic carbon adjacent to C=O) and *δ* 210 (carbonyl (C=O) carbon). IR spectrum of the compound showed the presence of primary O–H stretching at 1103 cm^−1^, 1165 cm^−1^ indicates carbon adjacent to keto group; 1381, 1465 cm^−1^ indicates CH stretching adjacent to C=O; 1735 cm^−1^ indicates C=O stretching in ketone; 2252, 2684, 2731, 2854 cm^−1^ represents C=C stretching; 2924 cm^−1^ indicates CH stretching in methylene group and 3150 cm^−1^ indicates N–H stretching of amino group. EI MS M/Z %the molecular formula of the compound BC 01_C1 was deduced as C_17_H_29_NO_3_ based on the results of elemental analysis (Anal Cal. for C_23_H_41_NO_5_: C 69.12%, H 9.89%, N 4.74%, 0 16.25%; Found C 70.02%, H 10.09%, N 5.14%, 0 14.75%) and in accordance with the number of carbon atoms observed on ^13^C NMR. The electron impact (EI) mass spectrum confirmed that the molecular weight of the antibiotic compound was 295.31. Based on the above spectral data by comparison with literature, the structure of the compound BC 01_C1 was established as 5-amino-2-(6-(2-hydroxyethyl)-3-oxononyl) cyclohex-2-enone and depicted in Fig. 1.

#### The compound BC 01_C2

**Fig. 2:**
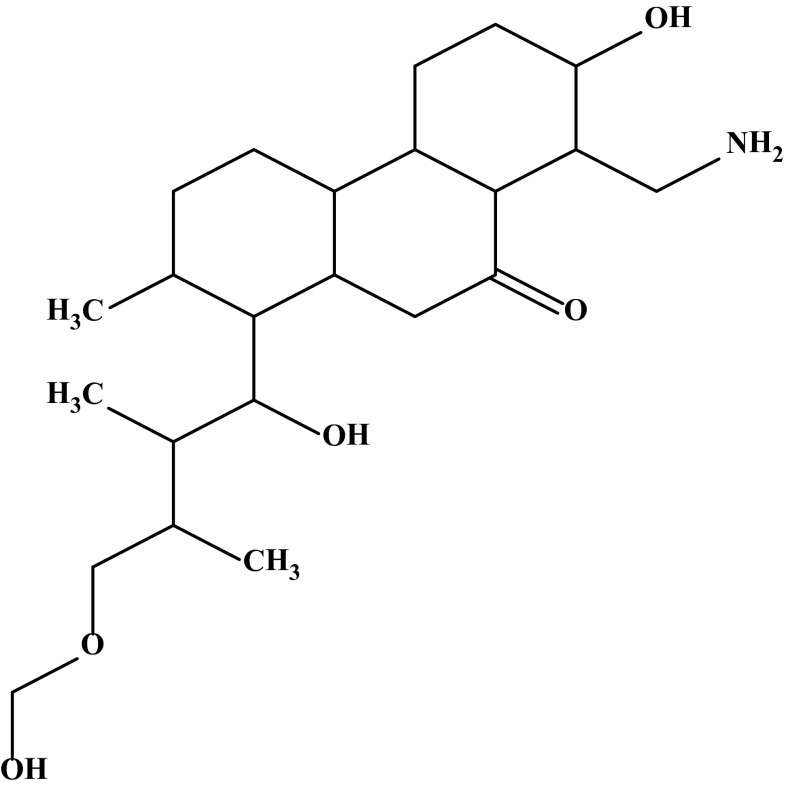
Structure of the compound BC 01_C2

The compound was isolated as a brown colour amorphous powder soluble in methanol, chloroform, ethyl acetate, benzene, acetone, ethanol, diethyl ether, DMSO and insoluble in hexane. The melting point of the compounds was found to be 401 °C. The purity of the newly isolated compound was evidenced by HPLC and found to be 98.89%. The UV spectrum of the purified active compound was displayed absorption maxima at 204 nm characteristic of phenanthrenone *λ*_max_ at 360 nm and 406 nm indicates the presence of hydroxyl and amino group respectively. ^1^H NMR (CDCl_3_: TMS 400 MHz) *δ* 0.977 (CH_3_ proton); *δ* 1.0–1.832 (CH protons in cyclohexane ring); *δ* 2.04–2.31 (CH protons attached to –C=O group); *δ* 2.29 (CH protons in cyclohexane attached to –CH_2_NH_2_ group); *δ* 3.49 (CH_2_ protons attached to –CH_2_–O–CH_2_ linkage); *δ* 3.574 (OH protons attached to cyclohexane); *δ* 3.645 (OH protons attached to –CH_2_–O–CH_2_ linkage); *δ* 5.011 (NH_2_ protons); *δ* 5.345 (CH_2_ protons). ^13^C NMR (CDCl_3_: TMS400 MHz) *δ* 14.31 (CH_3_ carbons); *δ* 22.88–32.116 (cyclohexane carbons attached to CH_3_ group); *δ* 76.88 (CH_2_ carbon attached to CH_2_–O–CH_2_ group); *δ* 77.2–77.5 (cyclohexane carbons); *δ* 100.31 (CH_2_ carbon attached to OH group); *δ* 211.5 (C=O carbon).IR spectrum of the compound showed 1111.0, 1172.72, 1373.32, 1465.90, 1820 cm^−1^ indicates (COOme); 3100 and 3150 cm^−1^ indicates (N–H stretching). EI MS M/Z %: the molecular formula of the antibiotic compound BC 01_C2 was deduced as C_23_H_41_NO_5_ based on the results of elemental analysis (Anal Cal. for C_23_H_41_NO_5_: C 67.12%, H 10.04%, N 3.40%, 0 19.44%; Found C 68.14%, H 11.24%, N 2.90%, 0 17.72%) and in accordance with the number of carbon atoms observed on ^13^C NMR. The electron impact (EI) mass spectrum confirmed that the molecular weight of the compound was 411.2. Based on the above spectral data by comparison with literature, the structure of the compound BC 01_C2 was established as 8-(aminomethyl)-7-hydroxy-1-(1-hydroxy-4-(hydroxylmethoxy)-2,3-dimethylbutyl)-2-methyldodecahydro phenanthren-9(1H)-one and shown in Fig. 2.

#### The compound BC 01_C3

**Fig. 3:**
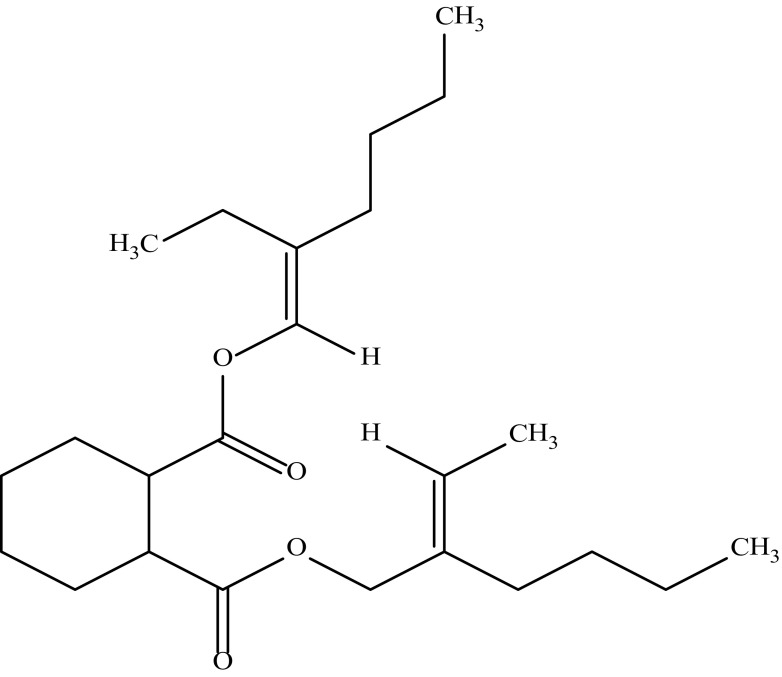
Structure of the compound BC 01_C3

The compound was isolated as a pale yellow colour amorphous powder soluble in methanol, chloroform, ethyl acetate, diethyl ether, ethanol, acetone, benzene, DMSO and insoluble in hexane. The melting point of the compounds was found to be 136.3 °C. The purity of the newly isolated compound was evidenced by HPLC and found to be 99.34%. The UV spectrum of the purified compound displayed absorption maxima at 203 nm indicates the presence of carboxylate group, 207 nm indicates the presence of cyclohexane-1,2-dicarboxylate and 360 nm indicates the presence of ethylidenehexyl group. ^1^H NMR (CDCl_3_: TMS 400 MHz) *δ* 0.94 (CH_3_proton); *δ* 1.29–1.48 (CH_2_ protons attached to CH_3_ group); *δ* 1.51 (CH protons in cyclohexane); *δ* 2.05 (CH_3_ protons attached to –C=C–H group); *δ* 2.804 (CH protons in cyclohexane); *δ* 5.37 (–C=C–H proton); *δ* 6.8 (–C=C–H proton attached to ester linkage). ^13^C NMR (CDCl_3_: TMS 400 MHz) *δ* 11.60 (CH_3_ attached to ethylene carbons); *δ* 22.8–32.11 (CH_2_ carbon adjacent to CH_3_ group); *δ* 69.04 (CH_2_ carbon attached to ethylene group); *δ* 114.25 (C=O carbons); *δ* 173.08 (CH_2_ protons adjacent to CH_2_–O–CH_2_ linkage); *δ* 173.50 (–COO-carbon). IR spectrum of the compound showed 779.24, 910.40 cm^−1^ (–C–O stretching), 1018.41, 1103.28, 1165.00, 1373.32, 1465.90, 1597.06 cm^−1^ (C–O–C) group); 1735.93 cm^−1^(C=O) group stretching); 2283.72, 2468.88, 2677.20, 2731.20, 2854.65, 2924.09 cm^−1^ (C–H stretching in esters). The molecular formula of the compound BC 01_C3 was deduced as C_24_H_40_O_4_ based on the results of elemental analysis (Anal Cal. for C_24_H_40_O_4_: C 73.43%, H 10.27%, O 16.30; Found C 74.2%, H 11.3%, 0 14.5%) and in accordance with the number of carbon atoms observed on ^13^C NMR. The electron impact (EI) mass spectrum confirmed that the molecular weight of the antibiotic was 392.29. Based on the above spectral data by comparison with literature, the structure of the compound BC 01_C3 was established as 1-((E)-2-ethylhex-1-en-1-yl)2-((E)-2-ethylidenehexyl) cyclohexane-1,2-dicarboxylate and shown in Fig. 3.

### Minimum inhibitory concentration (MIC)

The isolate BC 01 (crude) exhibits the maximum antibacterial activity against *P. vulgaris* (zone of inhibition 38 mm), whereas moderate activity was observed against *E. coli* (35 mm), *B. subtilis* (35 mm), *S. aureus* (32 mm), *P. aeruginosa* (32 mm) and *B. cereus* (29 mm). For antifungal studies, the isolate BC 01 exhibits highest activity against *A. niger* (28 mm) and showed moderate activity against *C. albicans* (19 mm), *A*. *flavus* (18 mm) *and S. cerevisiae* (14 mm) (Raghava Rao et al. 2012).

The purified compounds showed broad spectrum of antimicrobial activity against both gram-positive, gram-negative bacteria and also fungi. The compounds obtained from *S. coelicoflavus* BC 01 were BC 01_C1, BC 01_C2 and BC 01_C3. The MIC of three compounds was shown in Table 1. The MIC of the compound BC 01_C1 was identified as 25 µg/ml for* S. aureus*, *P. aeruginosa* and* P. vulgaris* whereas *E. coli* and *B. cereus* were inhibited at 50 µg/ml and *B. subtilis* at 75 µg/ml. In case of fungi the MIC was 100 µg/ml for *C. albicans* and *A. niger,* whereas 75 µg/ml for *A. flavus* and 50 µg/ml for *S. cerevisiae.* The MIC of the compound BC 01_C2 was observed at 12.5 µg/ml for *S. aureus* and *P. vulgaris*. At 25 µg/ml concentration *E. coli* was inhibited, whereas *B. Subtilis*, *B. cereus* and *P. aeruginosa* were inhibited at 50 µg/ml concentration. For fungal studies the MIC value was observed at 75 µg/ml for *A. niger*, *S. cerevisiae* and 100 µg/ml for *A. flavus* and 125 µg/ml for *C. albicans.* The MIC of the compound BC 01_C3 was observed at 12.5 µg/ml for *E. coli*, and in case of *S. aureus*, *B. cereus*, *P. aeruginosa* and *P. vulgaris* at 25 µg/ml, whereas for *B. subtilis* at 50 µg/ml. In case of fungal studies the MIC value for *A. niger* and *S. cerevisiae* was 50 µg/ml, whereas *A. flavus* was inhibited at 100 µg/ml and *C. albicans* at 125 µg/. From the above observations it was evident that the compounds BC 01_C1, BC 01_C2 and BC 01_C3 were acting as potent antibiotics against both the bacteria and fungi.

**Table 1:** Minimum inhibitory concentrations of three compounds

Name of the organism	Name of the compound		
	BC 01_C1 (µg/ml)	BC 01_C2 (µg/ml)	BC 01_C3 (µg/ml)
*S. aureus* (MTCC 3160)	25	12.5	25
*B. subtilis* (MTCC 441)	75	50	50
*B. cereus* (MTCC 430)	50	50	25
*P. aeruginosa* (MTCC 424)	25	50	25
*E. coli* (MTCC 443)	50	25	12.5
*P. vulgaris* (MTCC 426)	25	12.5	25
Fungi			
*C. albicans* (MTCC 227)	100	125	125
*A. niger* (MTCC 961)	100	75	50
*A. flavus* (MTCC 3396)	75	100	100
*S. cerevisiae* (MTCC 170)	50	75	50

The MIC of the three compounds BC 01_C1, BC 01_C2 and BC 01_C3 were tested against a wide variety of test organisms for which the MIC values ranged from 12.5 to 75 µg/ml for bacteria and 50 to 125 µg/ml for fungi. Similar investigations and results were obtained by Smaouia et al. (2011) where they reported three pure bioactive compounds named compound 1 (a diketopiperazine derivative), compound 2 (a phthalate derivative) and compound 3 (a cyclic tetrapeptide derivative) from *Streptomyces* sp. TN17 strain. These three active compounds possess potent antibacterial and antifungal activities. Likewise on purification of *Streptomyces* sp. Strain US80 led to isolation of three pure active compounds. The chemical structures of these three compounds are named as irumamycin (1a), X-14952B (1b) and17-hydroxy-venturicidin A (1c), which possess potent antimicrobial activities against gram-positive, gram-negative bacteria and fungi (Fguira et al. 2005). Similar studies were carried out by Mehdi et al. (2006) in which the strain *Streptomyces* sp. TN97 produces three compounds, which are diketopiperazine derivatives, isocoumarin derivative and the *N*-acetyl-tyramine exhibits potent antimicrobial activities against gram-positive, gram-negative bacteria and fungi (Mehdi et al. 2006) and also compounds 5,7-dimethoxy-4-*p*-methoxylphenylcoumarin and 5,7-dimethoxy-4-phenylcoumarin isolated from *S. aureofaciens* CMUAc130 exhibited antifungal activities against tested fungi, and their MICs were found to be 120 and 150 µg/ml respectively and also two pure active compounds were isolated from *Streptomyces* sp. US24 possessed antibacterial activity against Gram-positive and Gram-negative bacteria (Mellouli et al. 2003).

### In vitro antioxidant activity

In the present study, in vitro antioxidant activity of the three isolated compounds was evaluated by DPPH free radical scavenging, the ferric reducing power (FRAP) assay and total antioxidant capacity (TAC). Ascorbic acid was used as positive control. All the assays were carried out at concentrations of 5, 10, 15 and 20 μg/ml and the results were expressed as average of three independent experiments.

The DPPH free radical scavenging activity was expressed as % of inhibition. Among the three compounds, BC 01_C3 (63.44 ± 0.67) exhibits the maximum inhibition of DPPH free radical scavenging activity followed by BC 01_C2 (61.92 ± 6.95) and BC 01_C1 (52.39 ± 1.68), respectively, at 20 µg/ml of concentration. All the three compounds possess significant free radical scavenging activity when compared with reference standard ascorbic acid. ANOVA analysis showed that there was a significant variation of DPPH free radical scavenging activity between the three compounds BC 01_C1, BC 01_C2 and BC 01_C3. According to Duncan’s grouping analysis there was a significant difference within each group at various concentrations. Among, these BC 01_C1 showed significant variation at concentrations of between 5, 15 and 20 µg/ml and also a significant variation between 10 and 20 µg/ml concentrations. Compound BC 01_C2 exhibited significant variation between 5, 15 and 20 µg/ml, whereas BC 01_C3 showed significant variation for all the four concentrations. The results are tabulated in Table 2.

**Table 2:** DPPH activity of the three compounds

% Inhibition				
Concentration of the compounds (µg/ml)	Name of the compounds			
	BC 01_C1*	BC 01_C2*	BC 01_C3*	Ascorbic acid*
5	34.81 ± 5.56a	36.82 ± 2.12a	36.38 ± 2.12a	47.03 ± 15.9a
10	39.25 ± 4.90ab	43.69 ± 1.42a	47.01 ± 0.87b	51.24 ± 7.31a
15	44.95 ± 4.07bc	52.03 ± 2.02b	55.74 ± 4.16c	67.96 ± 25.69b
20	52.39 ± 1.68c	61.92 ± 6.95c	63.44 ± 0.67d	70.64 ± 25.07b
	*F* = 9.29**	*F* = 23.87**	*F* = 81.64**	*F* = 0.921@

* Each value represents mean ± SD of three independent experiments; ^@^ Not significant

** The values represent the means (±SD) of three independent experiments. Means within a column followed by the same letter are not significantly different from each other at *p* = 0.05 according to Duncan’s multiple range test (DMRT)

The ferric reducing antioxidant power was expressed as μmoles/ml of ascorbic acid equivalents. All the three compounds possess worthy activity when compared with the reference standard ascorbic acid; among the three compounds, BC 01_C1 (60.00 ± 4.00) exhibited higher ferric reducing antioxidant activity followed by BC 01_C3 (59.53 ± 3.00) and BC 01_C2 (56.66 ± 2.51), respectively, at 20 μg/ml of concentration. According to the ANOVA analysis there was significant variation of ferric reducing antioxidant power with respect to ascorbic acid equivalents among the three compounds BC 01_C1, BC 01_C2 and BC 01_C3. Duncan’s grouping analysis showed that there was a significant variation within each group at various concentrations. Among these, BC 01_C2 showed significant variation of all the four concentrations whereas BC 01_C1 showed at concentrations, of between 5µg/ml, 15 µg/ml, and 20 µg/ml was also a significance variance between 10 and 20 µg/ml. Compound BC 01_C3 exhibited significance variance between 5, 10 and 15 µg/ml, but not 15 and 20 µg/ml. The results are tabulated in Table 3.

**Table 3:** FRAP activity of the three compounds

Ascorbic acid equivalents (μmoles/ml)				
Concentration of the compounds (µg/ml)	Name of the compounds			
	BC 01_C1*	BC 01_C2*	BC 01_C3*	Ascorbic acid*
5	42.00 ± 5.00a	38.56 ± 1.42a	41.50 ± 1.32a	25.33 ± 2.51a
10	46.33 ± 4.04ab	43.30 ± 1.60b	47.83 ± 1.19b	42.66 ± 2.51b
15	52.66 ± 2.08bc	48.16 ± 3.68c	55.83 ± 2.32c	53.33 ± 3.21c
20	60.00 ± 4.00c	56.66 ± 2.51d	59.53 ± 3.00c	69.33 ± 4.50d
	*F* = 11.95**	*F* = 29.19**	*F* = 44.62**	*F* = 94.64**

* Each value represents mean ± SD of three independent experiments

** The values represent the means (±SD) of three independent experiments. Means within a column followed by the same letter are not significantly different from each other at *p* = 0.05 according to Duncan’s multiple range test (DMRT)

The total antioxidant capacity activity was expressed as μm/ml of ascorbic acid equivalents. Increase of the absorbance indicated the increase of the total antioxidant capacity. Among the three compounds the TAC was found to be highest for BC 01_C2 (67.83 ± 24.79) followed by BC 01_C1 (67.00 ± 29.51), BC 01_C3 (60.00 ± 22.98), respectively, at a concentration of 20 μg/ml. The compounds possess worthy total antioxidant capacity activity when compared with the standard ascorbic acid. According to the ANOVA analysis, there was a significant variation of total antioxidant capacity observed for the compounds BC 01_C1 and BC 01_C2, but in case of BC 01_C3, there was no significant variation. Duncan’s grouping analysis showed that there was a significant variation for compounds BC 01_C1 and BC 01_C2 at concentrations of among 5, 15 and 20 µg/ml and also a significant variation between 10 and 20 µg/ml. For compound BC 01_C3 exhibited significant variation between 5 and 20 µg/ml but not between 10µg/ml, 15 µg/ml, and 20 µg/ml. The results are tabulated in Table 4.

**Table 4:** Total antioxidant capacity of the three compounds

Ascorbic acid equivalents (μmoles/ml)				
Concentration of the compounds (µg/ml)	Name of the compounds			
	BC 01_C1*	BC 01_C2*	BC 01_C3*	Ascorbic acid*
5	15.33 ± 2.08a	12.90 ± 0.17a	13.66 ± 9.23a	19.33 ± 5.50a
10	29.66 ± 11.50a	18.73 ± 4.81a	30.00 ± 19.92ab	33.00 ± 10.81b
15	44.00 ± 19.69ab	49.36 ± 30.54ab	51.16 ± 32.68ab	56.33 ± 12.66c
20	67.00 ± 29.51b	67.83 ± 24.79b	60.00 ± 22.98b	71.33 ± 17.03d
	*F* = 4.17**	*F* = 5.13**	*F* = 2.52@	*F* = 10.86**

* Each value represents mean ± SD of three independent experiments; ^@^ Not significant

** The values represent the means (±SD) of three independent experiments. Means within a column followed by the same letter are not significantly different from each other at *p* = 0.05 according to Duncan’s multiple range test (DMRT)

Crude extract of BC 01 showed significant DPPH radical scavenging activity 68.91 ± 21.00, and FRAP activity 78.00 ± 15.10 ascorbic acid equivalents and total antioxidant activity 93.33 ± 2.52 ascorbic acid equivalents at a concentration of 20 µg/ml (Rao and Rao 2013).

It is essential to use more than one method to evaluate the antioxidant activity of isolated compounds. The mechanism involved in antioxidant activity is the ability to donate a hydrogen atom to a radical, and the propensity for donating hydrogen is a critical factor in free radical scavenging (Bondet et al. 1997). The DPPH free radical scavenging assay has been widely used to evaluate antioxidant capacities. Antioxidants react with DPPH, reducing a number of DPPH molecules equal to the number of available hydroxyl groups (Matthaus 2002). The degree of discoloration indicates that the samples to scavenge free radical due to its ability to donate hydrogen proton this shows that when the concentration of the compound increased the free radical scavenging activity also increases. All the three compounds possess worthy activity but the compound BC 01_C3 exhibited potent DPPH free radical scavenging activity and TAC when compared with other two compounds. The FRAP assay is commonly used in routine analysis for evaluation of antioxidant capacity. In the present study the compound BC 01_C1 exhibited potent ascorbic activity equivalents when compared with other two compounds. The reducing capacity of a compound might serve as a significant indicator of its potential antioxidant capacity. FRAP assay measures the reducing capability of tested sample by increasing sample absorbance based on the ferrous ions released (Prior et al. 2005). Similar type of study was conducted by Saurav and Kannabiran (2012) and reported a compound 5-(2,4-dimethylbenzyl) pyrrolidin-2-one isolated from marine *Streptomyces* VITSVK5 exhibited potent the DPPH radical scavenging and phosphomolybdenum reduction activities (44.13 and 50.10% at 5 µg/ml, respectively). In another study, Kumaqai et al. (1993) reported a compound PC-766 B isolated from *Nocardia brasiliensis* exhibited dose-dependent antioxidant activity. In addition, the compounds 2-allyoxyphenol (Arumugam et al. 2010) and streptopyyrolidine isolated from *Streptomyces* were reported to possess antioxidant activity (Shin et al. 2008). Likewise, Kim et al. (2008) isolated a compound Protocatechualdehyde from *Streptomyces lincolnensis* M-20 that exhibited potent antioxidant activity, and also meromonoterpene compounds like Cymopo and avrainvilleol isolated from marine sponges exhibited potent antioxidant activity (Takamatsu et al. 2003).

### In vitro anti-inflammatory activity by HRBC membrane stabilization method

The purified compounds were examined for their in vitro anti-inflammatory activity by HRBC membrane stabilization method. Stabilization of the RBCs membrane was studied to establish the mechanism of anti‐inflammatory action of three compounds extracted from *Streptomyces coelicoflavus* BC 01. The anti-inflammatory activity was expressed as % of inhibition. Among the three compounds, the % of inhibition was found to be highest for BC 01_C3 (82.86 ± 12.47) followed by BC 01_C1 (73.89 ± 12.50) and BC 01_C2 (71.26 ± 2.53), respectively, at their highest concentration, 20 μg/ml. All the three compounds possess potent anti-inflammatory activity when compared with the standard Diclofenac sodium. According to the ANOVA analysis there was a significant variation of anti-inflammatory activity that was observed for the compounds BC 01_C1 and BC 01_C2, but in case of BC 01_C3 no significant variance was observed. In Duncan’s grouping analysis there was a significant variation observed for BC 01_C1 and BC 01_C2 at concentrations of among 50, 100 and 200 μg/ml but no significant difference was observed in between 100 and 200 μg/ml. In case of compound BC 01_C3 no significant variation was observed for all the concentrations and the results are tabulated in Table 5.

**Table 5:** Anti-inflammatory activity of the three isolated compounds

% of inhibition				
Concentration of the compounds (µg/ml)	Name of the compounds			
	BC 01_C1*	BC 01_C2*	BC 01_C3*	Diclofenac sodium*
50	36.79 ± 9.74a	56.79 ± 1.13a	50.23 ± 20.48a	62.96 ± 7.01a
100	57.51 ± 2.93b	67.65 ± 4.67b	67.51 ± 19.94a	76.17 ± 4.73b
200	73.89 ± 12.50b	71.26 ± 2.53b	82.86 ± 12.47a	84.35 ± 3.66b
	*F* = 11.96**	*F* = 17.23**	*F* = 2.46@	*F* = 12.01**

* Each value represents Mean ± SD of three independent experiments; ^@^ Not signficant

** The values represent the means (±SD) of three independent experiments. Means within a column followed by the same letter are not significantly different from each other at *p* = 0.05 according to Duncan’s multiple range test (DMRT)

The compounds effective in inhibiting the hypotonic solution induced haemolysis at different concentrations. The three compounds may possibly inhibit the release of lysosomal content of neutrophils at the site of inflammation. These neutrophil lysosomal constituents include bactericidal enzymes and proteases, which upon extracellular release cause further tissue inflammation and damage (Chou 1997). The haemolytic effect of hypotonic solution is related to excessive accumulation of fluid within the cell resulting in the rupturing of its membrane. Such injury to RBC membrane will further render the cell more susceptible to secondary damage through free radical-induced lipid peroxidation (Ferrali et al. 1992). Compounds with membrane stabilizing properties are well known for their ability to interfere with the early phase of inflammatory reactions, namely the prevention of the release of phospholipases that trigger the formation of inflammatory mediators (Aitadafoun et al. 1996). In the present study the three compounds isolated from *S. coelicoflavus* BC 01 showed potent anti-inflammatory activity. Earlier studies also revealed that the compounds 5,7,4′-trimethoxy-4-phenylcoumarin and 5,7-dimethoxy-4-phenylcoumarin produced from *Streptomyces aureofaciens* CMUAc130 possess in vitro anti-inflammatory activity (Taechowisan et al. 2007). A polyether compound, dianemycin, isolated from *Streptomyces* sp. MT 2705-4: KCTC 8651P, showed a potent anti-inflammatory activity (Lee et al. 1997), and also five new anti-inflammatory activity compounds, Phaeochromycins A-E, were isolated from the *Streptomyces phaeochromogenes* LL-P018 (Graziani et al. 2005). For findings the anti-inflammatory compounds from *Streptomyces,* a compound lansai C extracted from *Streptomyces* sp. SUC1exhibits potent in vitro anti-inflammatory activity (Taechowisan et al. 2007). Three peptides, Salinamide C, E and D isolated from marine *Streptomyces* sp. CNB-091 act as anti-inflammatory peptides. These metabolites are useful as antibiotic and anti-inflammatory agents (Moore et al. 1999). Cyclomarin A is a new cyclic heptapeptides antibiotic isolated from *Streptomyces* sp. It exhibited significant anti-inflammatory activity in both in vivo and in vitro assays (Renner et al. 1999). Consequently, the compound isolated from Streptomyces coelicoflavusBC 01might be a natural source of membrane stabilizer and capable of providing an alternative remedy for the management and source of membrane stabilizer.

In our search for antibiotic producers from mangrove soil the isolate *Streptomyces coelicoflavus* BC 01 was capable of producing antibiotics, the antibiotics generated by *Streptomyces coelicoflavus* BC 01 is a primary bioactive constituent isolated from the culture filtrate, after conducting the separation and purification procedures its structure was determined by using spectroscopic techniques. Neither the proposed molecular structure of the isolated three antibiotic compounds, nor its molecular weight, resembles any of the antibiotics generated by any of the strains of *Streptomyces* species. Finally, by conducting a search using chemspider as well as a survey of the available literature, the structure of the antibiotic generated by this local strain in the present investigation proved not to be identical with any other reported antibiotics. The antibiotic compounds evidenced an in vitro antimicrobial activity against gram-positive and gram-negative bacteria, as well as fungi and also exhibit in vitro antioxidant and anti-inflammatory activity.
